# Why do orthopaedic surgeons get sued? An analysis of £2.2 billion in claims against NHS England: trends in litigation and strategies to enhance care

**DOI:** 10.1007/s00402-025-05957-y

**Published:** 2025-06-21

**Authors:** Saran Singh Gill, Kapil Sugand, Chinmay Madhukar Gupte

**Affiliations:** 1 Imperial College London, London, UK; 2 Royal National Orthopaedic Hospital, London, UK; 3 Imperial College Healthcare NHS Trust, London, UK

**Keywords:** Orthopaedics, Litigation, Malpractice, Legal, NHS

## Abstract

**Background:**

Litigation in Orthopaedic Surgery poses a significant financial challenge to healthcare systems. Orthopaedic-related claims accounted for 10.8% of the 10,900 total claims in the NHS in 2023/24, costing approximately £250 million. Yet, no extended analysis of Orthopaedic-related litigation trends has been conducted. This study examined NHS litigation data from 1996/97 to 2023/24 identifying trends, causes, and financial impact to provide actionable insights for improving clinical practice.

**Methods:**

Orthopaedic-related claims data from NHS Resolution (NHSR; 1996/97–2023/24) were analysed under the Freedom of Information Act. The dataset, focused on closed claims with settlements, included causes, injury types, and payouts. Broader classifications were applied due to GDPR constraints. Non-parametric distributions were confirmed using the Shapiro-Wilk test. Subsequent analyses, using the Kruskal-Wallis tests, calculated significant differences between categories and across years.

**Results:**

Between 1996/97 and 2023/24, 22,606 clinical negligence claims resulted in 14,702 settlements exceeding £2.2 billion, including £1.2 billion in damages. Musculoskeletal injuries were most frequent primary injuries (21%, £407.53 million), followed by unnecessary operations and postoperative pain (22%, £328.27 million). Neurological issues (8%) and poor outcomes (13%) accounted for £254.74 million and £129.14 million, respectively. Surgical errors (24%) caused the highest damages of the primary causes (£309.47 million), followed by failure or delayed treatment (23%, £277.02 million) and decision-making errors (22%, £287.57 million). Settlement values peaked in the early 2010s before declining, with significant differences in median claims, damages, and total payouts per annum (*p* < 0.001).

**Conclusion:**

Between 1996/97 and 2023/24, over £2.2 billion was paid in settlements, with £1.2 billion in damages. Musculoskeletal injuries, surgical errors, and delayed treatment were leading causes, highlighting persistent clinical challenges. Although claim volumes and payouts have declined since 2011/12, improved consent and multidisciplinary meetings may offer potential opportunities to enhance patient outcomes and reduce litigation against Orthopaedic Surgeons in the NHS.

**Supplementary Information:**

The online version contains supplementary material available at 10.1007/s00402-025-05957-y.

## Introduction

Litigation remains a significant challenge in Orthopaedic Surgery worldwide [1, 2]. In the UK, NHS Resolutions (NHSR), formerly the NHS Litigation Authority, now oversees both clinical and non-clinical claims, employing a robust framework for monitoring and auditing departmental and individual performances across the NHS [3, 4]. Through these targeted strategies, NHSR aims to alleviate the pressures of litigation, with a strong emphasis on achieving resolutions outside of court, thereby reducing the financial and operational burden of litigation within the NHS [5]. While the NHS indemnifies its physicians, with staff covered by state-backed Clinical Negligent Scheme for Trusts (CNST) via NHSR, Claoué has noted the emergence of a “complaints culture”, fuelled by patient‑empowerment initiatives like the Patient Advice and Liaison Service (PALS), which despite its role in advocating for patients, may inadvertently drive higher rates of litigation [6–10]. As such, a longitudinal analysis of the successful complaints received is required to understand the reasons for claims, and work to improve the service as a whole.

 In 2023/24, the NHS faced 10,900 claims overall, with Orthopaedic Surgery accounting for 10.8% of these, one of the highest among all specialties, down from 12% in 2019/20 [ 5, 11, 12 ]. The financial impact of these Orthopaedic-related claims alone reached approximately £250 million for the year [ 5, 13 ]. Prior studies by Atrey et al. and Majeed et al. analysed Orthopaedic-related claims within the NHS, reporting cumulative claim values of over $300 million from 2000 to 2006 and £1.2 billion from 2008 to 2019, respectively [ 14, 15 ]. The predominant causes of these claims were related to issues of mismanagement, diagnostic errors, perioperative complications and consent, with these finding being echoed in the current literature [ 14 – 19 ]. Yet, no studies to date have analysed the litigation outcomes over the past 25 years.

## Aims

To examine the NHSR database for Orthopaedic Surgery-related claims between 1996/97 and 2023/24, categorising those claims, analysing trends over time, and identifying key areas for improvement in clinical practice and risk management.

## Methods

Data on clinical claims related to Orthopaedic Surgery in England from the financial years 2006/07 to 2022/23, where the incident date is between financial years 1996/97 and 2023/24, was obtained from NHSR under the Freedom of Information Act [20, 21]. This retrospective dataset included information on each claim, such as the primary cause, type of injury, incident year, settlement year, associated damages and legal costs. Analysis was restricted to closed claims with financial settlements, considering only completed payouts.

Given the structure of the NHS Resolution database, due to adherence with the Data Protection Act 2018 and General Data Protection Regulation (GDPR) laws, claims could not be categorised by specific Orthopaedic subspecialties [22, 23]. Instead, claims were grouped into broader categories based on primary cause and injury type, allowing for generalised yet meaningful classification. Data were further stratified by systems affected and primary reasons for claims [14]. Analysis focused on total payouts, both inclusive and exclusive of legal fees, and longitudinal trends. Descriptive statistics, including median and IQR were calculated using RStudio (RStudio Team, 2024). Descriptive statistics, including median and interquartile range (IQR), were calculated using RStudio (RStudio Team, 2024). Non-parametric distributions were confirmed via the Shapiro-Wilk test. Longitudinal differences were assessed using the Kruskal-Wallis test, with statistical significance set at *p* < 0.05.

## Results

**Fig. 1:**
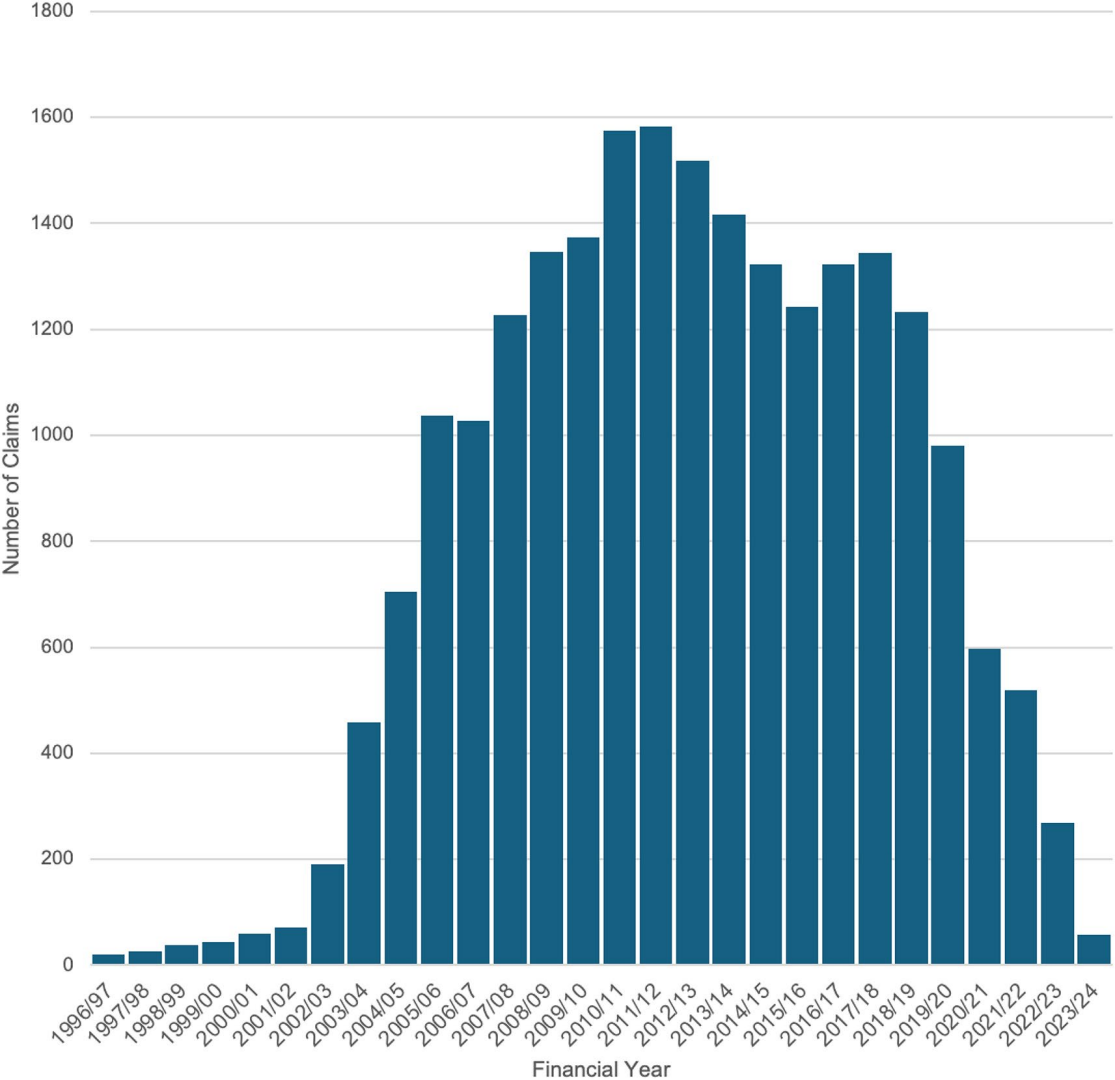
Number of clinical claims and incidents received number of clinical claims and incidents received between financial years ‘2006/07’ and ‘2023/24’ where the incident date is between financial years ‘1996/97’ and ‘2023/24’ and the Specialty is ‘Orthopaedic Surgery’ The bar chart displays the number of claims by year of incident, illustrating a clear trend over time. The x-axis represents the Year of Incident, ranging from 1996/97 to 2023/24, and the y-axis represents the Number of Claims

**Fig. 2:**
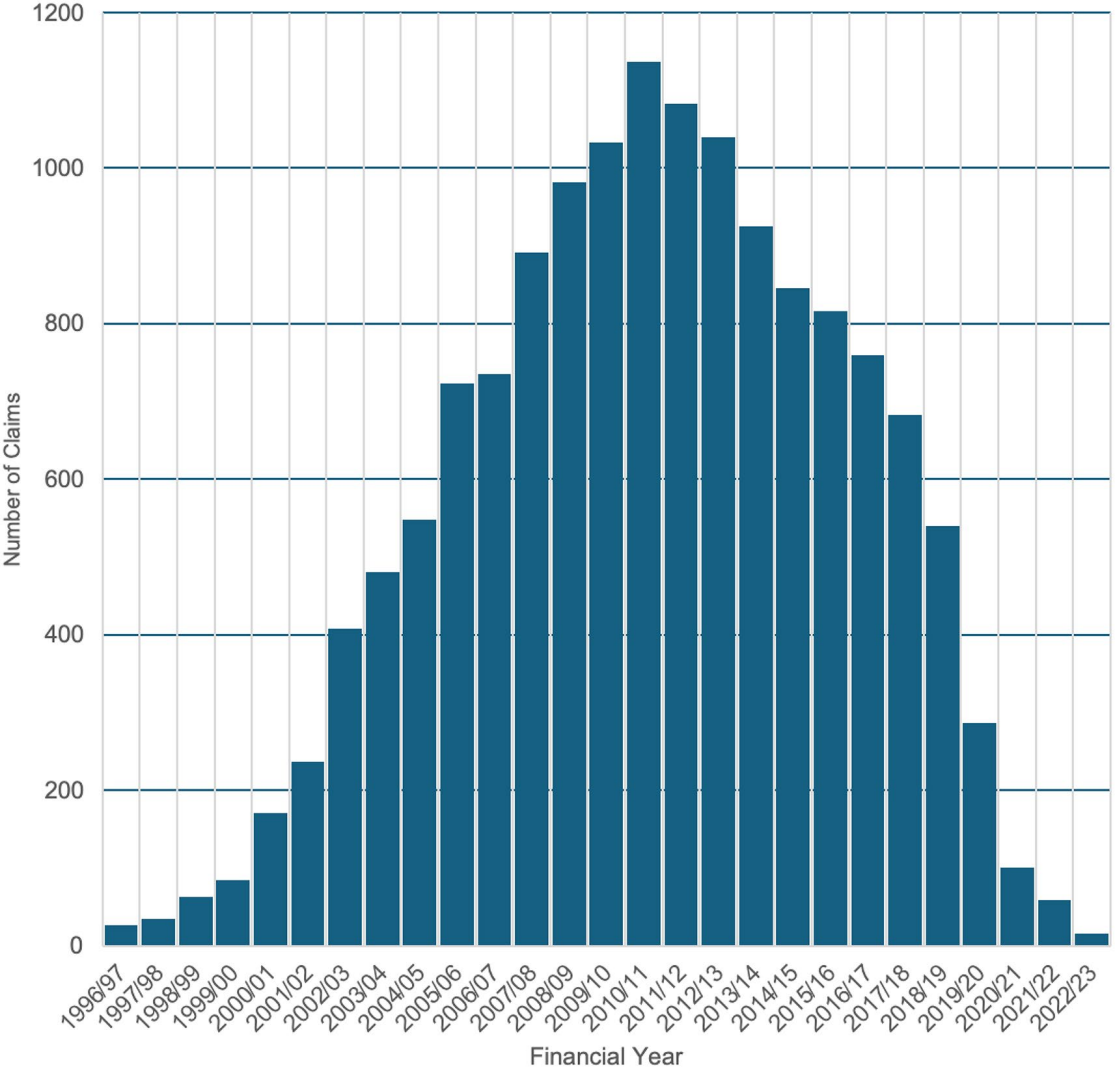
Number of clinical claims closed (or settled with a periodical payment order) number of clinical claims closed (or settled with a periodical payment order) between financial years ‘2006/07’ and ‘2022/23’ with a damages payment, and where the incident date is between financial years ‘1996/97’ and ‘2022/23’ and the Specialty is ‘Orthopaedic Surgery’. The bar chart shows the number of closed claims by year of incident, highlighting the distribution and trends in closed claims over time. The x-axis represents the Year of Incident, spanning from 1996/97 to 2023/24, while the y-axis represents the Number of Closed Claims

**Fig. 3:**
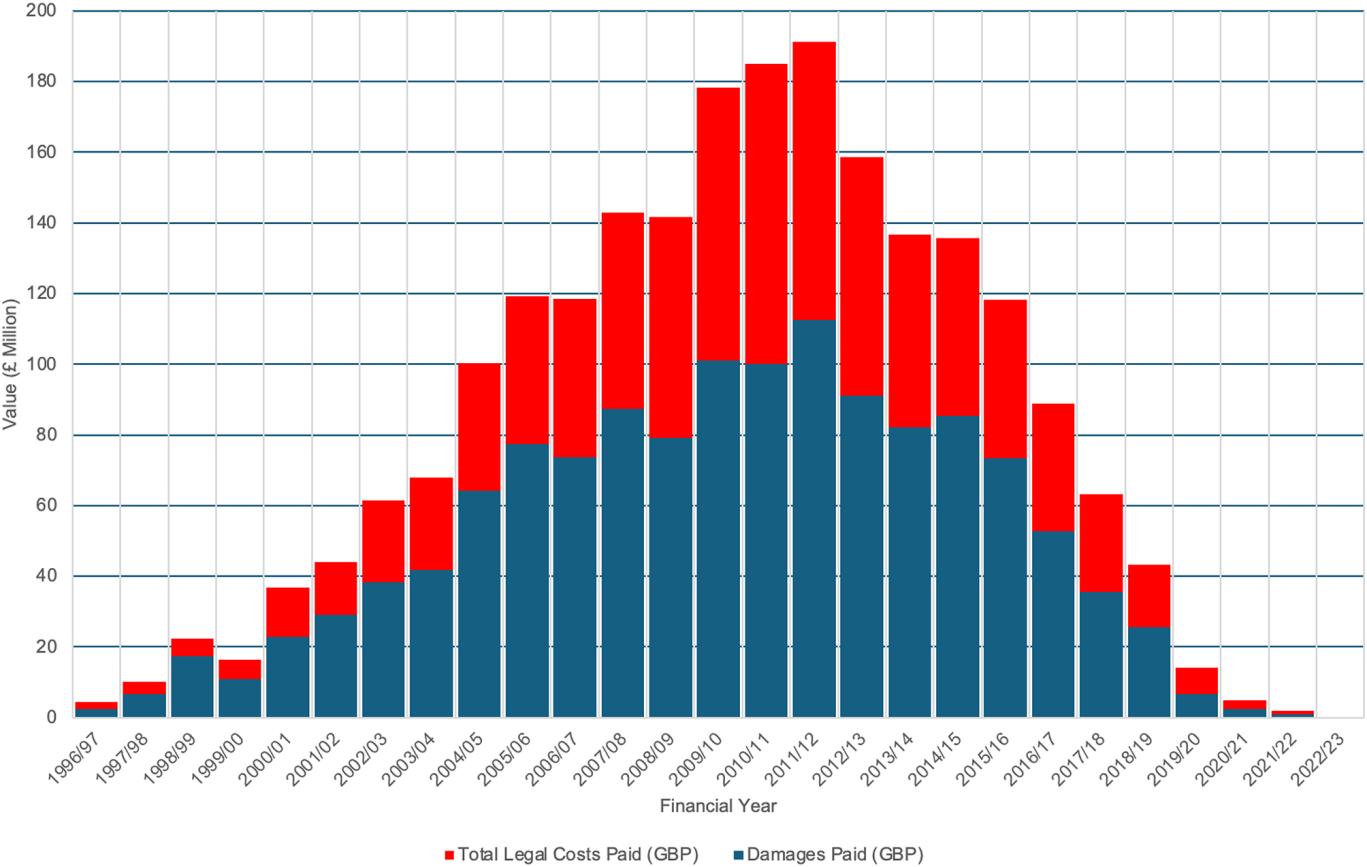
Cost of clinical claims closed (or settled with a periodical payment order) between financial years ‘2006/07’ and ‘2022/23’ with a damages payment, and where the incident date is between financial years ‘1996/97’ and ‘2022/23’ and the Specialty is ‘Orthopaedic Surgery’. The bar chart represents the total amount paid by year of incident, showing the financial trends related to claims over time. The x-axis depicts the Year of Incident, ranging from 1996/97 to 2023/24, and the y-axis shows the total paid in millions of GBP. The red series represents the total paid out in each year, and the blue represents the damages paid per year

**Fig. 4:**
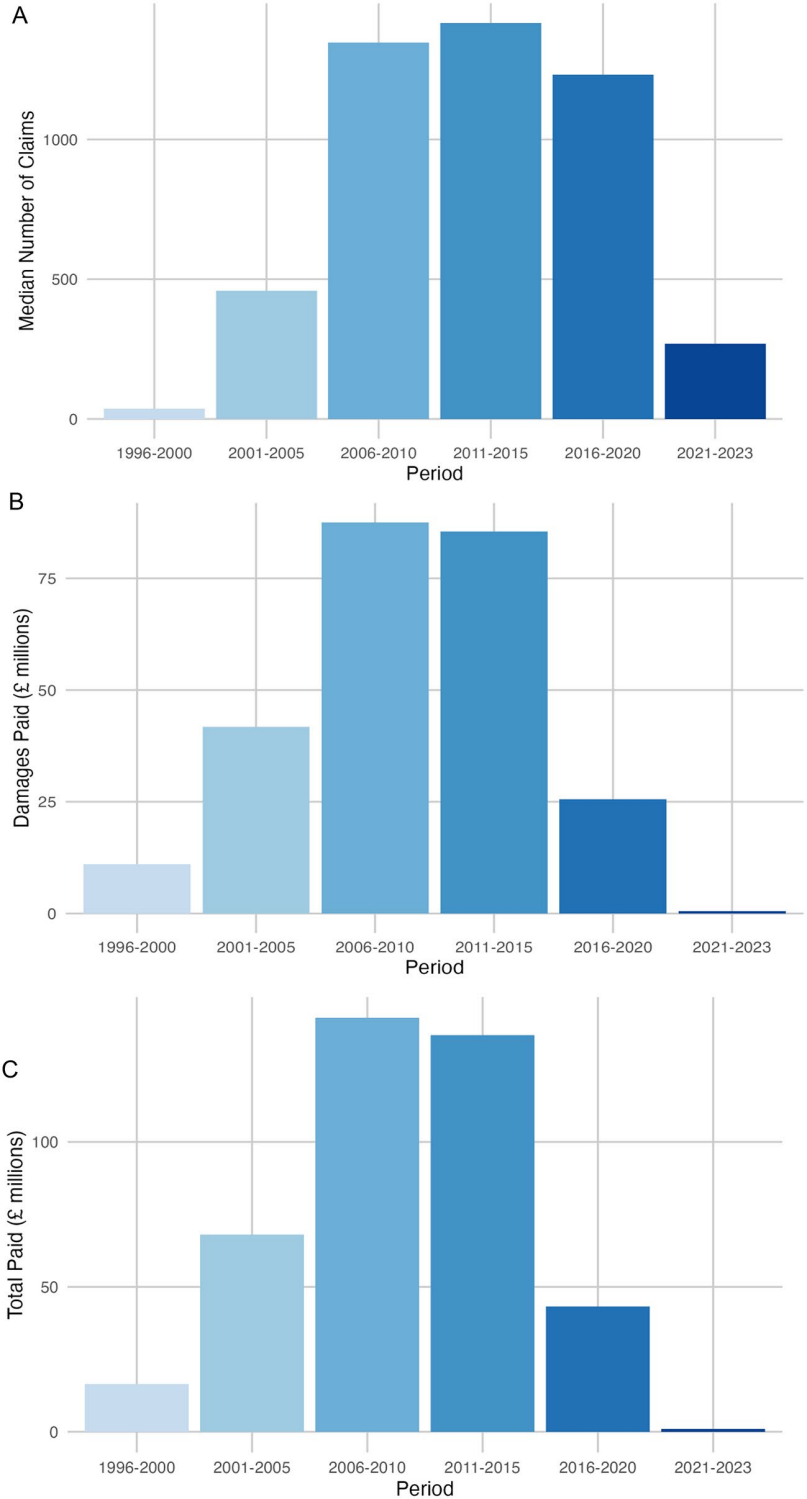
Trends in claims, damages paid, and total paid over 5-year periods. (**A**) Presents the median number of claims filed per 5-year period, with the x-axis representing the 5-year blocks and the y-axis showing the median number of claims. The black error bars represent the interquartile range (IQR), illustrating variability in claims filed within each period. This visualization helps assess whether the frequency of claims has increased, decreased, or remained stable over time. (**B**) Focuses on damages paid, scaled in millions of GBP, showing the median compensation amounts per 5-year period. The x-axis denotes the 5-year blocks, while the y-axis indicates the median damages paid. The IQR bars reflect fluctuations in compensation amounts, highlighting periods where damages payments exhibited greater variability. (**C**) Represents the median total amount paid, including damages and legal costs, for each 5-year period, also scaled in millions of GBP. The x-axis depicts the 5-year blocks, and the y-axis shows the median total paid. The black error bars indicate the IQR, emphasizing differences in total payouts across periods. This visualization captures financial trends in claims management, helping to assess the overall economic burden over time

Between 1996/97 and 2023/24, a total of 22,606 claims were recorded, of which 14,702 claims (65.0%) were resolved through financial settlements by 2022/23 (Figs. 1–3). Over this period, the total value of settlements exceeded £2.2 billion, with damages alone accounting for more than £1.2 billion (54.5%). Settlement values peaked notably in the early 2010s, culminating in a peak in 2011/12 (£112,497,517 in damages, £191,373,792 total paid out). This was followed by a gradual decline, including potential COVID-19 distortions, with settlements dropping to £234,880 in 2022/23 (£120,812 in damages). Significant differences were observed in the median number of claims per year, damages paid per year, and total payouts per year (*p* < 0.001) when analysed across 5-year periods (Fig. 4). A detailed breakdown of settlement values by injury type and claim reason was provided in Tables 1 and 2, with granular insights in Supplementary Tables 1 and 2.

**Table 1:** Top payouts per category, by type of injury. All percentages are given to the 1 decimal place, with any values less than 0.1 after formatting denoted as < 0.1. Any rows with less than 5 claims are denoted as ‘x’ due to GDPR related redactions from NHSR. The table is ordered in descending order based on claim volume

Category	No. of Claims (%)	Damages Paid in GBP (%)	Total Paid in GBP (%)
Additional/unnecessary Operation(s)	3,175 (21.7)	200,973,887 (15.3)	364,060,711 (16.6)
Musculoskeletal	3,133 (21.4)	407,533,680 (31.1)	632,465,732 (28.9)
Unnecessary Pain	2,640 (18.0)	127,291,139 (9.7)	242,722,414 (11.1)
Poor outcome	1895 (12.9)	129,142,972 (9.9)	234,427,837 (10.7)
Neurological	1,187 (8.1)	254,741,712 (19.4)	390,410,970 (17.8)
Skin and Tissue	976 (6.7)	26,906,065 (2.1)	55,917,789 (2.6)
Fatality	391 (2.7)	29,879,156 (2.3)	51,427,973 (2.4)
Circulatory and Cardiovascular	381 (2.6)	33,279,603 (2.5)	56,941,021 (2.6)
Infection	316 (2.2)	39,293,350 (3.0)	62,366,453 (2.9)
Gastrointestinal, Hepatic and Genitourinary	175 (1.2)	36,770,148 (2.8)	57,198,341 (2.6)
Other	142 (1.0)	440,6021 (0.3)	9,974,321 (0.5)
Oncological	68 (0.5)	9,848,261 (0.8)	14,815,385 (0.7)
Psychiatric	68 (0.5)	2,390,168 (0.2)	4,646,789 (0.2)
Multiple Systems	35 (0.2)	6167,971 (0.5)	9,490,460 (0.4)
Anaesthetic	24 (0.2)	293,416 (< 0.1)	747,774 (< 0.1)
Renal	19 (0.1)	1,310,269 (0.1)	2,389,567 (0.1)
Respiratory	19 (0.1)	387,053 (< 0.1)	977,460 (< 0.1)
Anaphylaxis	15 (0.1)	69,716 (< 0.1)	293,132 (< 0.1)
Developmental	X	X	X

**Table 2:** Top payouts per category by reason for claim. All percentages are given to the 1 decimal place, with any values less than 0.1 after formatting denoted as < 0.1. Any rows with less than 5 claims are denoted as ‘x’ due to GDPR related redactions from NHSR. The table is ordered in descending order based on claim volume

Category	No. of Claims (%)	Damages Paid in GBP (%)	Total Paid in GBP (%)
Surgical Error	3,249 (24.1)	309,474,849 (25.9)	502,928,548 (25.0)
Failure of/Delayed Treatment	3,132 (23.2)	277,016,946 (23.1)	471,380,401 (23.5)
Decision Making	3,003 (22.3)	287,572,742 (24.0)	484,486,177 (24.1)
Improper Observations/Care	2,033 (15.1)	119,208,691 (10.0)	207,983,981 (10.3)
Consent	1,192 (8.8)	139,984,534 (11.7)	233,903,711 (11.6)
Medication	212 (1.6)	12,939,258 (1.1)	21,516,047 (1.1)
Other	181 (1.3)	12,094,543 (1.0)	20,114,893 (1.0)
Infection	161 (1.2)	18,978,724 (1.6)	31,968,727 (1.6)
Unnecessary Surgery	143 (1.1)	9,468,237 (0.8)	17,806,116 (0.9)
Discharge	82 (0.6)	6,081,400 (0.5)	10,522,137 (0.5)
Administration	50 (0.4)	1,013,032 (0.1)	1,967,892 (0.1)
Misconduct	24 (0.2)	2,049,940 (0.2)	3,590,089 (0.2)
Communication	10 (0.1)	457,699 (< 0.1)	829,774 (< 0.1)
Death	7 (0.1)	422,956 (< 0.1)	924,274 (< 0.1)
Anaesthetic	X	x	x

### Closed claims by primary cause

Surgical errors were the most common cause of claims, accounting for 24% of cases (3,249 claims). Failure or delayed treatment closely followed, comprising 23% (3,132 claims), with decision-making errors representing 22% (3,003 claims). Improper observations or care contributed 15% of claims (2,033 cases). Other causes, such as administration errors, communication issues, consent problems, unnecessary surgery, infections, medication errors, misconduct, and discharge-related issues, collectively made up 14% of claims (946 cases), with each category contributing less than 2%.

Surgical errors also incurred the highest financial damages, accounting for 26% (£309.47 million). Decision-making errors contributed 24% (£287.57 million). Failure or delayed treatment represented 23% (£277.02 million). Improper observations or care accounted for 10% (£119.21 million).

**Fig. 5:**
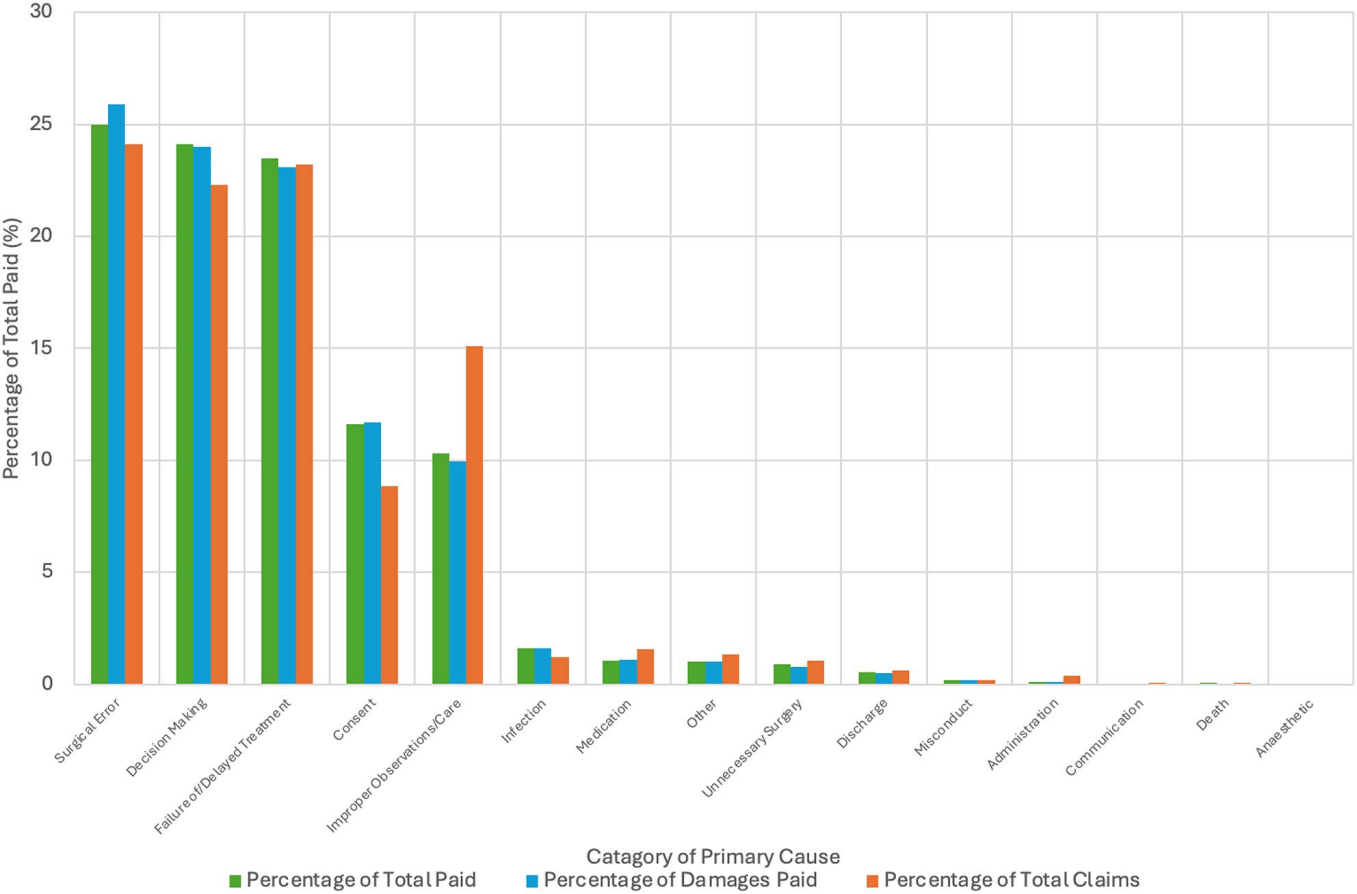
Percentage of total closed claims, total damages paid and total payout by category of primary cause. This figure represents the distribution of total closed claims by category, expressed as a percentage, ordered by proportion of total damages paid Categories with higher percentages have a larger share of claims in the dataset. supplementary Table 1 outlines the breakdown by individual cause

In total payouts, surgical errors represented 25% (£502.93 million), followed by decision-making errors (24%, £484.49 million) and failure or delayed treatment (23%, £471.38 million). Improper observations or care made up 10% (£207.98 million), with other causes collectively accounting for 18% (£362.05 million). These trends are summarised in Table 2 and visualised in Fig. 5.

### Closed claims by primary injury

Musculoskeletal injuries emerged as the most frequently reported primary injury type, accounting for 21% of claims (3,133 cases). Unnecessary operations and pain collectively made up 22% of claims (5,815 cases), while other significant categories included poor outcomes (13%, 1,895 cases) and neurological issues (8%, 1,187 cases).

Musculoskeletal injuries also accounted for the highest financial costs, representing 31% of damages (£407.53 million) and 29% of total payouts (£632.47 million). Neurological issues accounted for 19% of damages (£254.74 million) and 18% of total payouts (£390.41 million). Combined, unnecessary pain and additional operations contributed 25% of damages (£328.27 million) and 28% of total payouts (£606.78 million).

**Fig. 6:**
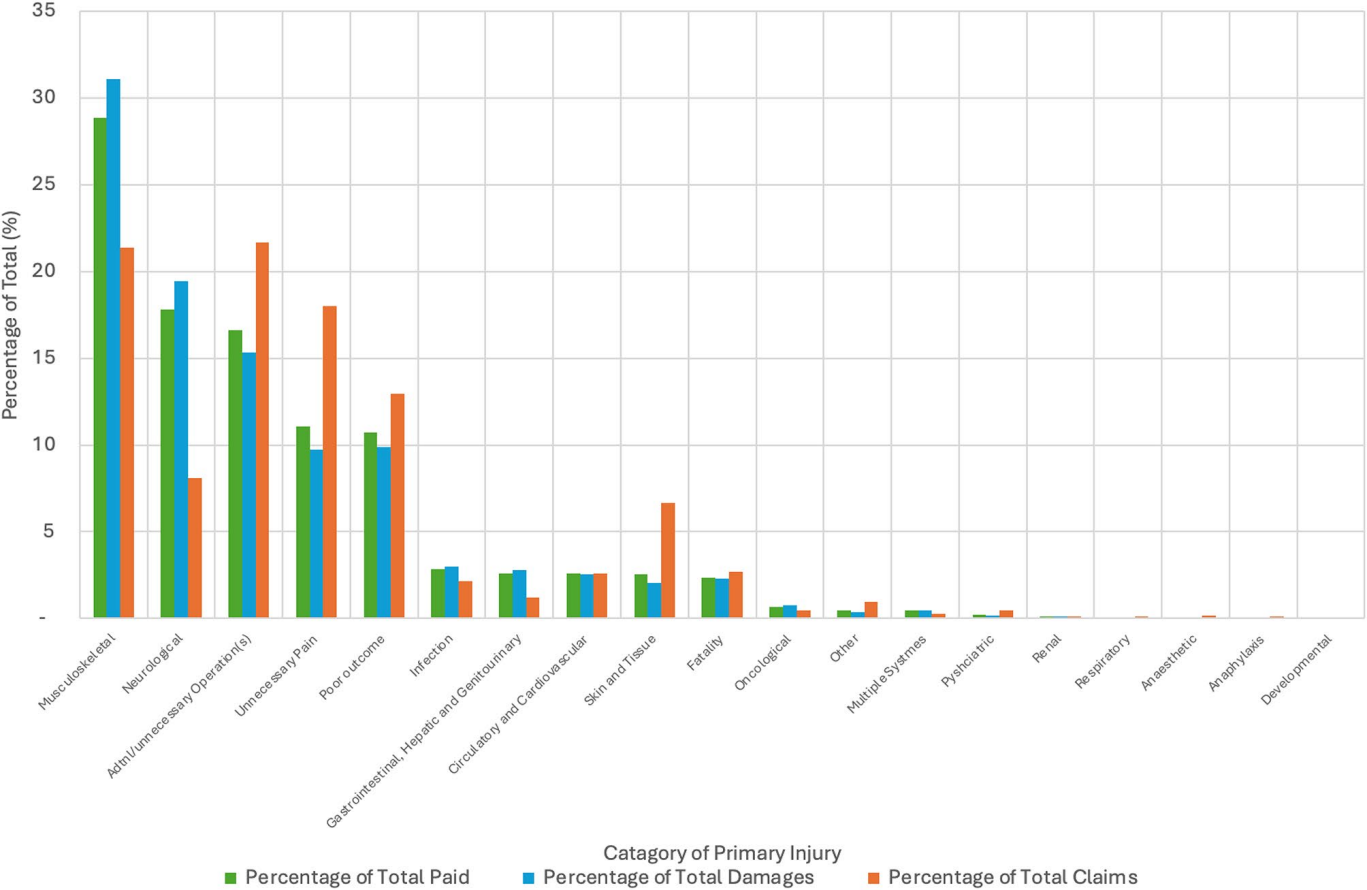
Percentage of total closed claims, total damages paid and total payout by category of primary injury. This figure represents the distribution of total closed claims by category, expressed as a percentage organised in descending order by proportion of total damaged paid. Categories with higher percentages have a larger share of claims in the dataset. During the data reorganisation process, 21 rows with redacted data were identified, supplementary Table 2 outlines the breakdown by individual injury

The distribution of claims and payouts demonstrates that musculoskeletal injuries, unnecessary operations, and neurological issues are key drivers of financial settlements in orthopaedic litigation. Poor outcomes and other injuries, although less frequent, still contribute significantly to overall costs. These findings are summarised in Table 1 and visualised in Fig. 6.

## Discussion

The average number of settled claims was 544 per financial year during the study period (1996/97 to 2023/24). The average pay out cost was approximately £149,660 per settled claim between 1996/97 and 2022/23, with £81,620 of that attributed to damages. During the first decade of the study claims gradually increased, peaking in 2010/11, before declining over the following eight years. A significant reduction in claims and payouts was observed post 2018/19, coinciding with the establishment of NHS Resolution in 2017/18 and its focus on early dispute resolution. The most recent data show continued declines, with 2022/23 recording the lowest figures. (Figs. 1–4).

While most orthopaedic surgeries are successful, with total knee replacement achieving patient satisfaction rates exceeding 80%, the risk of complications remains [24–29]. Surgeons are obligated by the duty of candour to disclose all intraoperative complications to patients, even when this may lead to legal consequences [30]. Although this study provides data on complication rates over a 27-year period, further analysis and contextualisation are necessary to fully interpret these findings.

### Longitudinal analysis

 Between 2000/01 and 2005/06, infections were the leading cause of orthopaedic surgery-related litigation, in the NHS resulting in settlements exceeding $321.7 million (USD) across 2,321 successful cases [ 15 ]. This reflects the surgical practices of the time, with subsequent improvements in theatre sterility, hospital hygiene, and infection prevention measures likely contributing to a decline in infection-related claims. Increased awareness of infection risks and greater legal literacy among patients may also have driven the earlier spike in claims. Our data indicates that infection-related claims have since decreased to under 2% of all claims, demonstrating significant advancements in surgical practices and post-operative care [ 31, 32 ]. Over the course of this study, initiatives such as Getting It Right First Time (GIRFT), introduced in 2012/13, appear to have improved care standards across the NHS. Trends observed in Figs. 1–3 indicate reductions in claim volumes and payouts from 2011/12 onwards [ 31 ]. In addition, initiatives such as Enhanced Recovery After Surgery (ERAS), though not directly analysed here, offer pathways to reduce litigation by improving postoperative care. By minimising complications, enhancing outcomes, and promoting faster recoveries, ERAS protocols align with broader efforts to enhance patient safety and satisfaction, potentially reducing litigation risks [ 33 ]. These findings underscore the importance of continuous quality improvement in healthcare delivery.

From 2005/06 to 2009/10, hip-related litigation decreased following the introduction of a best practice tariff by the Department of Health. Despite this, delays in diagnosis remained the leading cause of claims, accounting for 40% of cases, with delayed treatment rising from 15 to 23% pre- and post-guidelines [34]. Similarly, our data shows that failure or delayed treatment comprised 23% of claims (3,132 cases) and 23% of damages (£277.02 million), highlighting persistent challenges with timely diagnosis and treatment over the past 27 years, even amidst policy interventions. These findings suggest trust-based changes alone are insufficient, with further focus and resources required to address delays in care effectively through systemic and NHS spanning changes. Furthermore, Ahmed SS reported that errors in clinical management accounted for the highest number of closed claims, with 2,933 cases costing over £119 million between 2004/05 and 2013/14 [7]. Similarly, our data identifies decision-making errors as a major factor, contributing to 22% of claims (3,003 cases) and 24% of damages (£287.57 million). This underscores the enduring impact of clinical management challenges in orthopaedic litigation. Greater multidisciplinary input prior to interventions could mitigate these issues by promoting more comprehensive decision-making and reducing errors [35]. However, this must be balanced against the strain it places on the NHS, as increased collaboration requires additional time, resources, and coordination. Achieving this balance will be essential to improving outcomes while maintaining healthcare efficiency.

Majeed H et al. analysed 8,548 claims from 2008/09 to 2018/19, with payouts exceeding £1.2 billion, identifying mismanagement (39.0%), diagnostic issues (17.6%), and perioperative issues (15.9%) as the most common causes [14]. In contrast, our longitudinal dataset highlights surgical errors (24%), delayed treatment (23%), and decision-making errors (22%) as leading causes, with over £2.2 billion paid across 22,606 claims. Settlement values peaked in the early 2000s before declining in recent years, reflecting evolving claim patterns and rising financial pressures in orthopaedic litigation. Moreover, Chen et al. examined knee surgery claims from 2005/06 to 2009/10, finding total knee replacement accounted for 11.2% of closed cases, with payouts totalling £10.45 million, primarily linked to amputations, poor outcomes, incorrect prosthesis use, and infections [36]. In contrast, our broader dataset identifies musculoskeletal injuries, including amputations, as the most frequent injury type, comprising 21% of claims (3,133 cases) and 29% of total payouts (£632.47 million). Although our dataset lacks procedural granularity, this comparison underscores the sustained financial burden of orthopaedic-related complications, highlighting the need for innovative techniques and strategies to prevent operative injuries, improve outcomes, and reduce future claims.

### The drop in case volume in recent years

The growing public awareness of the financial, temporal and emotional hurdles involved in suing the NHS may to have tempered the overall volume of claims, potentially contributing to the reduction in claims demonstrated in this study [7, 49]. Moreover, the observed decline in closed claims may not reflect fewer incidents, since the dataset only captures cases concluded in court. Patients may face financial, geographic or informational barriers, long waits for expert reports or lack of awareness of compensation rights, which may deter valid claims [37]. In addition, The Legal Aid, Sentencing and Punishment of Offenders Act 2012 (LASPO) has reduced public funding for civil litigation, including clinical negligence cases in the UK, potentially preventing claimants from having access to funding that may have facilitated claims that are not straightforward [38, 39]. As such, this may play a role in the reduction in successful claim volume after 2012/13. In addition, this may prevent patients from submitting claims against actions that may yield a smaller magnitude of damages, likely reflecting both reduced access to and the tightening of pre action protocols that make small claims less viable [37]. Moreover, the three‑year statutes of limitation with the UK, alongside a growing need for more conclusive proof of injury or harm and expedited Advanced Dispute Resolution (ADR) pathways may cause meritorious cases to lapse before trial [40–42].

Yet, potentially due to the LASPO, the growth of no win no fee cases can lead to gouging of fees from legal fees, with successful cases receiving payments of up to 100% above normal rates, proving the be costly to the NHS [43, 44]. ADR, as such, could play a crucial role in preventing unnecessary litigation in the current climate, with Chia et al. finding that approximately 65% of hospitals in Taiwan resolved over 80% of medical disputes internally, without external mediation or litigation, potentially reducing the patient incentive to sue [45]. This approach has been mirrored by NHSR, with reported increases in mediation rates and a reduction in the rate of cases going to trial [46]. Lastly, changes in how claims are recorded or routed, such as diversion into mediation, can create the illusion of falling claim numbers even if patient harm remains constant, potentially skewing the interpretation of the results of our study [47, 48].

### COVID-19

Yet, the effect of the COVID-19 pandemic may have skewed the data in this study. During this period, acute orthopaedic trauma referrals declined by 34%, while mortality rates more than doubled to 3.8% (*p* < 0.01) [49]. As such, this may have affected perceptions of negligence and duty of care to patients, amid the media covering and lived experiences of the those suffering during the pandemic, with patients often willing to defer their surgeries due to COVID related anxiety [50]. Furthermore, consultants were twice as likely to act as the primary surgeon, introducing potential biases in clinical and legal outcomes that cannot be accounted for in our data, due to the lack of granularity in our dataset [49]. Similarly, the widespread suspension of elective procedures, with an estimated 91% cancellation rate, may have affected our results, as reflected in our data, with case volume dropping by over 50% between 2018/19 and 2019/20 [51]. Although this decline coincided with the implementation of NHSR, the precise impact of COVID-19 on orthopaedic litigation remains unclear. The suspension of elective surgeries likely worsened musculoskeletal conditions and patient dissatisfaction, potentially increasing medico-legal claims against orthopaedic surgeons. Understanding the impact of COVID-19 on orthopaedic care within the NHS is crucial for assessing future medico-legal risks, analysing evolving litigation patterns, and informing policy adaptations to address post-pandemic challenges. The significant backlog in orthopaedic procedures, estimated to require approximately £200 million to resolve, underscores the long-term consequences of service disruptions [52].

### Potential solutions and learning points: multidisciplinary team consensus in clinical management

Multidisciplinary approaches to the management of Orthopaedic patients have been shown to enhance postoperative outcomes in knee replacement, underscoring the value of multidisciplinary team (MDT) focussed care [53]. Our study found that decision-making errors accounted for 22% (3,003 claims) of all claims over the study period, highlighting the impact of suboptimal management. These findings align with existing literature, as Majeed et al. analysed 8,548 claims from 2008/09 to 2018/19, identifying mismanagement as a contributing factor in 39% of cases, with payouts exceeding £1.2 billion [14]. Similarly, Ahmed et al. reported that errors in clinical management accounted for the highest proportion of closed claims, with 2,933 cases resulting in costs exceeding £119 million between 2004/05 and 2013/14 [7]. Collectively, these studies underscored mismanagement as a persistent factor in litigation, reinforcing the necessity for enhanced clinical decision-making and structured MDT strategies to minimise associated risks.

Implementing an MDT-based approach optimises patient outcomes by reducing surgeon bias, promoting patient-centred care, and improving risk stratification to prevent misinterpretation of, or failure to, scan (accounting for over £32 million in damages, and £59 million total payout. Supplementary Table 2), and unnecessary procedures [54]. This collaboration minimises surgeon bias towards operating, ensures patient-centred decision-making, and reduces claims related to unnecessary surgeries [55–60]. Moreover, MDT discussions promote shared accountability and could be used to develop medicolegal protocols, as per Rougereau et al., which may reduce litigation related to surgical mismanagement [55–57, 61], while providing holistic evaluation of treatment options, optimising clinical outcomes, and reducing medicolegal risk [35, 62–65]. These factors cumulatively prioritise, uphold and enhance patient safety which is at the centre of clinical governance. Yet, to date, there is no literature available directly assessing the impact of MDT working on claim volumes. Future studies should aim to quantify the impact of MDT working on claim volume, increasing the evidence base for it.

### Potential solutions and learning points: preparation and planning for procedures

Comprehensive preparation and planning are essential for minimising litigation risks and optimising patient outcomes in surgical procedures [66, 67]. Our study identified 3,175 claims related to unnecessary or additional operations, costing the NHS over £230 million, with more than £140 million paid in damages. Enhanced preoperative planning, particularly in assessing potential complications, could help determine the necessity of surgical intervention and both prevent avoidable procedures while streamlining patients’ pathways to treatment, minimising delays in diagnosis and treatment (Supplementary Table 2) [55, 68–70]. While preoperative checklists have been shown to improve hazard detection, enhance communication, and reduce complications, their impact on lowering 90-day readmission rates remained uncertain [57, 71].

### Potential solutions and learning points: comprehensive consenting

Consent-related litigation remained a persistent challenge. £230 million, including £140 million in damages, was paid out for 1,192 orthopaedic claims related to consenting (Supplementary Digital Material: Table 2). This substantial financial burden underscored the need for improvements in the consenting process. Atrey et al. reported that between 2000 and 2006, these claims totalled £8.69 million, averaging £108,310 per case, despite representing only 3.37% of all cases [15]. These claims spanned both trauma and elective surgeries, arising mainly from failures to disclose recognised complications during the consent process.

Therefore, further analysis of the NHS consenting pathway is warranted, particularly regarding patient communication and understanding [72]. The British Orthopaedic Association (BOA) has endorsed the use of prewritten consent forms that are designed, via the OrthoConsent initiative in 2008, with patient input to reduce the rates of successful litigation against orthopaedic surgeons and trusts within the NHS [73]. These forms have been shown to improve patient understanding by around 25%, rising from 56.7% and 57.6–80.5% and 81.6%, respectively and may contribute to the reduction in claim volume beyond 2010/2011, accounting for a time lag in implementation but due to the nature of the dataset, this claim cannot be truly validated [74]. Yet, consent-related litigation remains an area of improvement in Orthopaedic surgery [75, 76].

In response, the use of visual aids has been proposed to improve the consenting process [77]. Sugand et al. conducted a randomised controlled trial concluding that patients considered visual aids as a key factor influencing satisfaction [78]. Anatomical models significantly enhanced patient satisfaction (*p* = 0.01), highlighting a cost-effective intervention with few barriers to implementation [78]. Moreover, digital tools and videos have been shown improve patient comprehension in 70% of studies, with methods such as videos, PowerPoints, and comprehension checkpoints proving effective for both satisfaction and comprehension based benchmarks [77, 79, 80]. This approach could reduce patient dissatisfaction and litigation claims, with a digital consent pathway estimated to save £201,590 per preventable claim [81]. Improving the consenting process by thoroughly explaining complications and adhering to the principles of Montgomery v. Lanarkshire Health Board ensures patients are fully informed. This could foster better decision-making, leading to improved patient satisfaction, subsequently reducing litigation risk [82].

### Potential solutions and learning points: duty of candour and governance

While complications are inevitable in clinical practice, the response to these incidents likely play a role in the subsequent process of litigation claims, an appropriate apology and competent investigation reducing the likelihood of claims being filed [83, 84]. Under the duty of candour, healthcare professionals must openly disclose when treatment does not go as planned, explain what happened, and outline measures to investigate and prevent recurrence [30, 85]. This transparency not only upholds patients’ right to make informed decisions but also builds trust, drives system‑wide learning and safety improvements, and by reducing the likelihood of surprise or mistrust, it can help mitigate the risk of future litigation and foster an culture of honesty and accountability [86, 87].

### Potential solutions and learning points: monitoring complications: morbidity and mortality meetings

Consistent monitoring of complications is key for Orthopaedic Surgeons to reflect on their current practice. This allows for the identification of areas for improvement, and subsequent implementation of evidence-based strategies to reduce complication rates and optimise surgical outcomes. Singh et al. proposed implementing monthly meetings focused on intra and postoperative morbidity and mortality for specialty trainees, providing a structured learning opportunity aligned with the WHO Patient Safety Curriculum [88, 89].

The findings suggest a positive early impact of these meetings, demonstrating improvements in patient safety knowledge, awareness, and attitudes towards patient safety among trainees [88]. This is particularly significant given our findings that surgical errors constitute the most common cause of litigation claims over the past 27 years, accounting for 24% of all closed cases. Structured discussions could enable Specialty Trainees to develop a deeper understanding of complications, equipping them with the skills necessary to implement safer practices. Furthermore, enhanced documentation and communication, reinforced through these meetings, may serve as a medico-legal safeguard, mitigating the risk of litigation resulting from errors or lapses in patient care [90–93].

### Potential solutions and learning points: registry reporting

Moreover, while Orthopaedic surgeries have high success and satisfaction rates, often exceeding 80%, the risk of complications remains [24–29, 94]. Surgeons are obligated by the duty of candour to disclose all intraoperative complications to patients, even when this may lead to legal consequences [30]. Transparent reporting mechanisms, such as the National Joint Registry (NJR), further support accountability and continuous quality improvement by systematically tracking surgical performance and complication rates [95–97]. By integrating these reporting structures with proactive learning initiatives, such as morbidity and mortality meetings, healthcare institutions can strengthen patient safety, enhance surgical outcomes, and reduce litigation risks.

### Future studies

Future studies should aim to develop a new anonymised NHSR litigation database by increasing the granularity and standardisation of claim data, enabling more precise analysis of trends and risk factors. Currently, GDPR constraints limit subspecialty classification, preventing detailed evaluations of procedure-specific claims [22, 23]. By categorising claims in accordance Orthopaedic procedures, potentially using OPCS or ICD codes, researchers could identify high-risk areas and develop targeted mitigation strategies.

Standardising the classification of claims across NHS trusts would also ensure consistency in identifying primary causes, distinguishing between surgical errors, diagnostic failures, and systemic factors such as resource limitations against claimant demographics. Additionally, integrating litigation data with clinical outcome registries, including the NJR, GIRIFT and HES data could provide deeper insights into the correlation between surgical complications and legal action, helping to refine surgical best practices [31, 33, 98, 99]. Expanding the dataset to include claims resolved through ADR and early-stage patient complaints would provide a more comprehensive picture of litigation trends, capturing disputes before they escalate to formal legal cases [42].

Complex procedures, particularly those involving high risk patients or later stage arthroplasty revisions, have been shown to benefit from the combined expertise of two senior surgeons and can yield improved intraoperative decision making, reduced complication rates and enhanced precision [100–103]. However, the impact of dual consultant operating on litigation rates and its cost‑effectiveness under the NHS model remain unknown and warrant investigation before it can be widely recommended.

### Limitations

This study evaluated NHS litigation data and payouts while addressing its inherent limitations. The dataset, obtained from NHS Resolution via a Freedom of Information request, was primarily collected for legal and financial purposes rather than research, which influenced its scope and utility. Furthermore, the dataset’s reliance on pre-defined categories posed inherent risks of misclassification, while the redaction of data for categories with fewer than five cases further limited the dataset, potentially leading to under-reporting. Informal case resolutions were also excluded, narrowing the dataset further.

Data was given in broad categories, preventing more granular statistical and thematic analysis of specific causes of litigation. A priori classification was employed to organise the data. However, claims such as surgical errors were categorised by primary cause rather than primary injury, as the dataset provided classifications for both, but lacked consistency in their application. These claims, reflecting patient perceptions of negligence, do not always align with actual clinical issues.

While the data offered valuable insights and identify areas for quality improvement, they do not represent a comprehensive measure of legal or clinical outcomes. Similarly, the effect of COVID-19 on these claims may have skewed outcomes after 2018/2019. Despite these limitations, this study provided a 27-year overview of Orthopaedic-related litigation trends, supporting efforts to reduce claims and enhance patient care.

## Conclusion

This study analysed orthopaedic litigation trends in the NHS from 1996/97 to 2023/24, revealing over £2.2 billion in settlements, including £1.2 billion in damages. Musculoskeletal injuries, surgical errors, and delayed treatment were the primary drivers of claims, reflecting persistent challenges in clinical management. Although claim volumes and settlement values have declined since 2011/12, significant opportunities remain to mitigate risks. Improving the consenting processes and consolidating a MDT-based approach to preoperative planning demonstrate potential to enhance patient outcomes and reduce litigation.

## Electronic supplementary material

Below is the link to the electronic supplementary material.

Supplementary Material 1

## Data Availability

No datasets were generated or analysed during the current study.
