# Supplementary material for: Why do orthopaedic surgeons get sued? An analysis of £2.2 billion in claims against NHS England: trends in litigation and strategies to enhance care
Source: Arch Orthop Trauma Surg. 2025 Jun 21;145(1):351. doi: 10.1007/s00402-025-05957-y (PMC12182531; doi:10.1007/s00402-025-05957-y)
Supplement: Supplementary file 1 — Supplementary Material 1 [file 402_2025_5957_MOESM1_ESM.docx]

## Supplementary digital content

| **Primary Injury** | **Category** | **No. of Claims** | **Damages Paid** | **Total Paid** |
| --- | --- | --- | --- | --- |
| Adtnl/unnecessary Operation(s) | Adtnl/unnecessary Operation(s) | 3,175 | 200,973,887 | 364,060,711 |
| Unnecessary Pain | Unnecessary Pain | 2,640 | 127,291,139 | 242,722,414 |
| Poor Outcome - Fractures Etc. | Poor outcome | 1,895 | 129,142,972 | 234,427,837 |
| Fracture | Musculoskeletal | 1,107 | 44,788,774 | 88,275,412 |
| Nerve Damage | Neurological | 867 | 126,094,341 | 206,777,996 |
| Joint Damage | Musculoskeletal | 787 | 80,166,899 | 132,858,615 |
| Pressure Sores | Skin and Tissue | 487 | 15,875,228 | 32,454,555 |
| Fatality | Fatality | 391 | 29,879,156 | 51,427,973 |
| Amputation - Lower | Musculoskeletal | 372 | 146,537,596 | 203,736,553 |
| Tendon Damage | Musculoskeletal | 236 | 9,102,108 | 18,866,951 |
| Thrombosis/Embolism | Circulatory and Cardiovascular | 233 | 11,992,200 | 22,962,171 |
| Limb Deformity | Musculoskeletal | 199 | 17,810,806 | 30,628,365 |
| Spinal Damage | Musculoskeletal | 195 | 84,563,674 | 118,942,744 |
| Burn(s) | Skin and Tissue | 189 | 2,841,197 | 6,325,159 |
| Other Infection | Infection | 186 | 26,071,270 | 40,126,727 |
| Scarring | Skin and Tissue | 178 | 1,871,935 | 5,477,029 |
| Foot Drop | Neurological | 165 | 24,085,612 | 41,252,016 |
| Dislocation | Musculoskeletal | 145 | 10,652,515 | 17,687,720 |
| Other | Other | 110 | 3,067,639 | 7,419,558 |
| Amputation - Upper | Musculoskeletal | 87 | 13,353,808 | 20,328,335 |
| Compartment Syndrome | Circulatory and Cardiovascular | 78 | 14,461,076 | 22,088,651 |
| Hospital Acquired Infection | Infection | 72 | 9,672,418 | 15,541,835 |
| Psychiatric/Psychological Dmge | Psychiatric | 68 | 2,390,168 | 4,646,789 |
| Tissue Damage | Skin and Tissue | 67 | 4,215,291 | 7,087,369 |
| Bladder Damage | Gastrointestinal, Hepatic and Genitourinary | 54 | 10,096,756 | 16,378,152 |
| Bowel Damage/ Dysfunction | Gastrointestinal, Hepatic and Genitourinary | 45 | 9,096,561 | 14,710,943 |
| Partial Paralysis | Neurological | 44 | 26,598,403 | 38,322,772 |
| Paraplegia | Neurological | 42 | 34,549,597 | 47,003,948 |
| Cosmetic Disfigurement | Skin and Tissue | 33 | 1,933,889 | 4,117,403 |
| Incontinence | Gastrointestinal, Hepatic and Genitourinary | 33 | 10,485,111 | 16,929,785 |
| Advanced Stage Cancer | Oncological | 33 | 3,867,059 | 6,444,542 |
| Rupture | Gastrointestinal, Hepatic and Genitourinary | 31 | 912,232 | 1,882,682 |
| Cancer | Oncological | 29 | 5,778,629 | 7,930,134 |
| Arterial Damage | Circulatory and Cardiovascular | 28 | 4,430,677 | 6,970,675 |
| Infectious Diseases | Infection | 26 | 2,181,133 | 3,883,109 |
| Infection (bacterial) | Infection | 25 | 895,585 | 2,031,599 |
| Cardiac Arrest | Circulatory and Cardiovascular | 23 | 1,586,390 | 2,736,243 |
| Multiple Injuries | Multiple Systmes | 22 | 1,199,181 | 2,469,737 |
| Bruising/ Extravasation | Skin and Tissue | 22 | 168,525 | 456,274 |
| Brain Damage | Neurological | 21 | 17,413,081 | 22,816,955 |
| Not Specified | Other | 21 | 937,645 | 1,825,552 |
| Respiratory Disorder/ Failure | Respiratory | 19 | 387,053 | 977,460 |
| Renal Damage/ Failure | Renal | 19 | 1,310,269 | 2,389,567 |
| Stroke | Neurological | 18 | 5,551,534 | 7,809,934 |
| Tetraplegia/ Quadriplegia | Neurological | 18 | 19,361,920 | 24,833,173 |
| Anaphylact Shock/Allergic Shock/allergy | Anaphylaxis | 15 | 69,716 | 293,132 |
| Multiple Disabilities | Multiple Systems | 13 | 4,968,790 | 7,020,723 |
| Dental Damage | Anaesthetic | 12 | 84,416 | 217,339 |
| Anaesthetic | Anaesthetic | 12 | 209,000 | 530,435 |
| Unknown | Other | 11 | 400,737 | 729,211 |
| Cardiovascular Condition | Circulatory and Cardiovascular | 8 | 408,603 | 847,850 |
| Sepsis | Infection | 7 | 472,944 | 783,183 |
| Perforation | Gastrointestinal, Hepatic and Genitourinary | 7 | 906,383 | 1,412,467 |
| Blindness | Neurological | 7 | 989,224 | 1,368,733 |
| Malignant Tumour | Oncological | 6 | 202,573 | 440,709 |
| Oedema | Circulatory and Cardiovascular | 6 | 139,621 | 629,479 |
| Aneurysm | Circulatory and Cardiovascular | 5 | 261,036 | 705,952 |
| Impotence | Gastrointestinal, Hepatic and Genitourinary | 5 | 5,273,105 | 5,884,312 |
| Osteoporosis | Musculoskeletal | 5 | 557,500 | 1,141,037 |
| Other Visual Problems | Neurological | 5 | 98,000 | 225,443 |
| Loss Of Sexual Function | Gastrointestinal, Hepatic and Genitourinary | x | x | x |
| Benign Tumour | Oncological | x | x | x |
| Reduced Life Expectancy | Other | x | x | x |
| Scalp Damage | Skin and Tissue | x | x | x |
| Partial Hearing Loss | Neurological | x | x | x |
| Epilepsy | Neurological | x | x | x |
| Decompression Illness | Other | x | x | x |
| Deafness | Neurological | x | x | x |
| Hemiparesis | Neurological | x | x | x |
| Addiction/Dependency | Other | x | x | x |
| Orthopaedic Injuries | Musculoskeletal | x | x | x |
| Loss Of Kidney | Renal | x | x | x |
| Liver Damage | Gastrointestinal, Hepatic and Genitourinary | x | x | x |
| Erb's Palsy | Neurological | x | x | x |
| Removal Of Cervix | Other | x | x | x |
| Lung Disease | Respiratory | x | x | x |
| Stunted Growth | Developmental | x | x | x |
| Spinabifida | Developmental | x | x | x |
| Cystic Growth | Developmental | x | x | x |
| Malnutrition | Other | x | x | x |
| Developmental Delay | Developmental | x | x | x |

Table 1 – incidence of, and total paid due to, closed claims by category of primary injury.

X represents data retracted by NHSR.

| **Primary Cause** | **Category** | **No. of Claims** | **Damages Paid** | **Total Paid** |
| --- | --- | --- | --- | --- |
| Fail / Delay Treatment | Failure of/Delayed Treatment | 3,132 | 277,016,946 | 471,380,401 |
| Failure/Delay Diagnosis | Failure of/Delayed Treatment | 2,047 | 207,872,461 | 340,034,779 |
| Intra-Op Problems | Surgical Error | 1,983 | 238,216,594 | 378,585,430 |
| Inappropriate Treatment | Decision Making | 1,332 | 126,033,620 | 214,171,896 |
| Operator Error | Surgical Error | 760 | 58,247,367 | 98,379,568 |
| Inadequate Nursing Care | Improper Observations/Care | 643 | 25,341,176 | 47,688,191 |
| Fail To Warn-Informed Consent | Consent | 604 | 61,373,302 | 106,694,797 |
| Fail To Recog. Complication Of | Consent | 588 | 78,611,232 | 127,208,914 |
| Failure To Interpret X-Ray | Improper Observations/Care | 365 | 18,303,764 | 33,691,772 |
| Delay In Performing Operation | Failure of/Delayed Treatment | 277 | 26,637,439 | 42,156,916 |
| Failure To X-Ray | Improper Observations/Care | 240 | 14,149,632 | 25,461,497 |
| Lack Of Assistance/Care | Improper Observations/Care | 183 | 6,345,034 | 12,493,024 |
| Fail To Follow-Up Arrangements | Improper Observations/Care | 181 | 7,147,362 | 14,133,340 |
| Failure To Perform Operation | Failure of/Delayed Treatment | 177 | 11,154,430 | 20,305,400 |
| Foreign Body Left In Situ | Surgical Error | 168 | 4,874,285 | 9,795,712 |
| Medication Errors | Medication | 145 | 8,005,774 | 13,694,318 |
| Perform. Of Op. Not Indicated | Unnecessary | 143 | 9,468,237 | 17,806,116 |
| Fail To Supervise | Decision Making | 137 | 9,659,580 | 15,197,930 |
| Failure To Perform Tests | Improper Observations/Care | 115 | 17,505,808 | 26,514,122 |
| Bacterial Infection | Infection | 106 | 13,337,743 | 22,944,660 |
| Lack Of Pre-Op Evaluation | Improper Observations/Care | 95 | 9,291,124 | 14,560,295 |
| Diathermy Burns/react. To Prep | Surgical Error | 93 | 1,709,995 | 3,599,149 |
| Fail To Carry Out PO Observs. | Improper Observations/Care | 93 | 10,811,423 | 16,671,051 |
| Equipment Malfunction | Other | 89 | 7,589,880 | 11,838,151 |
| Poor Application Of Plstr Cast | Surgical Error | 82 | 1,939,558 | 4,361,622 |
| Inappropriate Discharge | Discharge | 82 | 6,081,400 | 10,522,137 |
| Wrong Diagnosis | Decision Making | 80 | 7,448,394 | 12,464,913 |
| Inadequate Monitoring Intra-Op | Improper Observations/Care | 79 | 6,735,220 | 11,393,234 |
| Other | Other | 71 | 3,820,241 | 6,789,404 |
| Err With Agnt/Dose/Route/Selec | Medication | 67 | 4,933,484 | 7,821,729 |
| Fail To Act On Abnorm Test Res | Decision Making | 65 | 7,407,161 | 11,224,546 |
| Lack Of Facilities/Equipment | Administration | 50 | 1,013,032 | 1,967,892 |
| Wrong Site Surgery | Surgical Error | 46 | 1,744,669 | 3,197,211 |
| Wrong Site Surgery (Never Event) | Surgical Error | 38 | 1,071,865 | 1,777,519 |
| Op. On Wrong Patient/Body Part | Surgical Error | 35 | 394,948 | 696,973 |
| Failed Infection Control Policy/Hospital Hygiene | Infection | 29 | 4,569,998 | 6,703,663 |
| Fail/Delay Referring To Hosp. | Failure of/Delayed Treatment | 28 | 4,518,172 | 7,338,308 |
| Cross Infection | Infection | 26 | 1,070,983 | 2,320,404 |
| Application Of Excess Force | Misconduct | 24 | 2,049,940 | 3,590,089 |
| Inapprop. Case Selection | Decision Making | 23 | 6,716,697 | 9,686,287 |
| Incorrect Injection Site | Improper Observations/Care | 15 | 384,365 | 817,928 |
| Fail/Delay Admitting To Hosp. | Decision Making | 15 | 2,519,535 | 3,554,365 |
| Surg Foreign Body Left In Situ | Surgical Error | 13 | 288,658 | 476,144 |
| Premature Ceasure Of Treatment | Decision Making | 11 | 1,101,809 | 2,770,936 |
| Retained Instrument Post-Operation | Surgical Error | 10 | 119,500 | 321,282 |
| Fail To Infrm Test Rslts | Communication | 10 | 457,699 | 829,774 |
| Not Specified | Other | 9 | 503,047 | 843,652 |
| Retained Instrument Post-Operation (Never Event) | Surgical Error | 8 | 92,250 | 199,337 |
| Improp. Delegation To Junior | Decision Making | 8 | 652,326 | 1,243,408 |
| Wrong Site Surgery | Surgical Error | 8 | 545,594 | 1,216,669 |
| Infusion Problems | Improper Observations/Care | 8 | 89,800 | 231,255 |
| Unexpected Death | Death | 7 | 422,956 | 924,274 |
| Unknown | Other | 7 | 119,099 | 397,287 |
| Re-Canalisation | Improper Observations/Care | 6 | 314,500 | 566,040 |
| Inadqte Monitor In Recov Room | Improper Observations/Care | 5 | 294,984 | 589,061 |
| Wrong Site Surgery | Surgical Error | 5 | 229,566 | 321,932 |
| Fail To Interpret USS | Improper Observations/Care | 5 | 2,494,499 | 3,173,171 |
| Fail/Delay Avail Op Thtre | Failure of/Delayed Treatment | 5 | 287,100 | 690,192 |
| Injured By Another Patient | Other | 5 | 62,276 | 246,399 |
| Intubation Problems | Anaesthetic | x | x | x |
| Tooth Inj & Patient Posit Prob | Anaesthetic | x | x | x |
| Assault, Etc By Hospital Staff | Misconduct | x | x | x |
| Retained Instrument Post-Operation | Surgical Error | x | x | x |
| Problem Blood Fluids | Improper Observations/Care | x | x | x |
| Probs With Medical Records | Administration | x | x | x |
| Fail Mon Dose/rate Syntocinon | Medication | x | x | x |
| ECT Treatment | Other | x | x | x |
| Self Harm | Other | x | x | x |
| Failed Sterilisation | Failure of/Delayed Treatment | x | x | x |
| MisplacedNaso/OrogastricTubeNotDet | Failure of/Delayed Treatment | x | x | x |

Table 2 – incidence of , and total paid due to, closed claims by category of primary reason

X represents a redacted number, defined as less than 5.
